# Novel DNA methylation marker discovery by assumption‐free genome‐wide association analysis of cognitive function in twins

**DOI:** 10.1111/acel.13293

**Published:** 2021-02-02

**Authors:** Afsaneh Mohammadnejad, Mette Soerensen, Jan Baumbach, Jonas Mengel‐From, Weilong Li, Jesper Lund, Shuxia Li, Lene Christiansen, Kaare Christensen, Jacob V. B. Hjelmborg, Qihua Tan

**Affiliations:** ^1^ Unit of Epidemiology, Biostatistics and Biodemography Department of Public Health University of Southern Denmark Odense Denmark; ^2^ Unit of Human Genetics Department of Clinical Research University of Southern Denmark Odense Denmark; ^3^ Department of Clinical Biochemistry and Pharmacology Odense University Hospital Odense Denmark; ^4^ Computational Biomedicine Department of Mathematics and Computer Science University of Southern Denmark Odense Denmark; ^5^ Chair of Experimental Bioinformatics TUM School of Life Sciences Technical University of Munich Munich Germany; ^6^ Population Research Unit Faculty of Social Sciences University of Helsinki Helsinki Finland; ^7^ Digital Health & Machine Learning Research Group Hasso Plattner Institute for Digital Engineering Potsdam Germany; ^8^ Department of Clinical Immunology Copenhagen University Hospital Rigshospitalet Copenhagen Ø Denmark

**Keywords:** Cognitive function, epigenetics, generalized correlation coefficient, linear regression, twins

## Abstract

Privileged by rapid increase in available epigenomic data, epigenome‐wide association studies (EWAS) are to make a profound contribution to understand the molecular mechanism of DNA methylation in cognitive aging. Current statistical methods used in EWAS are dominated by models based on multiple assumptions, for example, linear relationship between molecular profiles and phenotype, normal distribution for the methylation data and phenotype. In this study, we applied an assumption‐free method, the generalized correlation coefficient (GCC), and compare it to linear models, namely the linear mixed model and kinship model. We use DNA methylation associated with a cognitive score in 400 and 206 twins as discovery and replication samples respectively. DNA methylation associated with cognitive function using GCC, linear mixed model, and kinship model, identified 65 CpGs (*p* < 1e‐04) from discovery sample displaying both nonlinear and linear correlations. Replication analysis successfully replicated 9 of these top CpGs. When combining results of GCC and linear models to cover diverse patterns of relationships, we identified genes like *KLHDC4*, *PAPSS2*, and *MRPS18B* as well as pathways including focal adhesion, axon guidance, and some neurological signaling. Genomic region‐based analysis found 15 methylated regions harboring 11 genes, with three verified in gene expression analysis, also the 11 genes were related to top functional clusters including neurohypophyseal hormone and maternal aggressive behaviors. The GCC approach detects valuable methylation sites missed by traditional linear models. A combination of methylation markers from GCC and linear models enriched biological pathways sensible in neurological function that could implicate cognitive performance and cognitive aging.

## Graphical abstract

The relationship between molecular profiles and cognitive function is complex. Through performance comparison with linear models and combination of results from the generalized correlation coefficient (GCC) and linear models, we were able to capture more CpGs of nonlinear association with cognition which could implicate in cognitive aging. This study promotes the use of GCC as a complementary method along with linear models in covering diverse patterns of relation to detecting more markers with cognition.

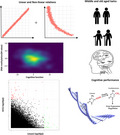

## 1 INTRODUCTION

Cognitive impairment refers to when a person has trouble remembering, learning new things, concentrating, or making decisions that can affect one's everyday life. Cognitive impairment in the elderly is costly and a key issue for health and social care since aging is the greatest risk factor for it. The level of cognitive functioning is shown to decrease with age accompanied by increased variability in twin pairs (McCartney et al., 1990). Epigenetic modification such as DNA methylation is a promising marker in understanding many age‐related phenotypes (Bakulski & Fallin, 2014). Despite the wide‐spread performance of epigenetic association studies, limited markers have been detected. The limitation in marker detection might have different reasons such as the distribution of the phenotype of interest which is not always normal and the complex patterns of relationship between epigenetic markers and cognition, possibly involving any non‐random patterns not limited to linearity. The multiple assumptions in the conventional statistical models could be responsible for the low replication and limited explanation in the phenotype variation by the identified markers.

Monozygotic (MZ) twins are valuable for controlling the genetic background in identifying epigenetic associations, as they share similar genetic makeups. In addition, differences in DNA methylation levels between discordant monozygotic twins associated with mental disorders have been frequently discussed (Castellani et al., 2015). However, modeling the correlation in dependent samples like twins requires additional assumptions concerning covariance structure and degree of genetic relatedness.

As an alternative, assumption‐free measurements of association or generalized correlation coefficient (GCC) have been proposed for omics studies (Murrell et al., 2016; Reshef et al., 2011). We think that this method has advantages to be considered for analyzing epigenetic data as the associations between methylation values and cognitive function are expected to be complex and more importantly, this method does not rely on strict assumptions such as normality of phenotypes and linear correlation.

In this study, we aim at promoting the use of GCC as a complementary method along with traditional linear models. We here investigate the performances of popular conventional models and the GCC method in an epigenome‐wide association study on cognitive function using DNA methylation data measured in blood samples of 400 MZ twins as a discovery sample. Performing single CpG sites differentially methylated regions (DMRs) and pathway analyses. Also, top single CpG sites and candidate regions in nearby genes are replicated in cognitive function using an independent DNA methylation sample of 206 twins (192 MZ and 14 dizygotic (DZ) twins) and verified in a gene expression data, respectively.

## 2 RESULTS

The descriptive statistics of the study samples are shown in Table S1. DNA methylation data for the entire sample passed the quality control (QC) were explained in the method section. We observed significant associations of cognitive measurement scores with age and sex in the middle‐aged Danish twin (MADT) sample (Pedersen et al., 2019). The cognitive score declines with age (*p* = 1.9e‐05) and is higher in females in comparison to males (*p* = 0.003). Additionally, none of the cell counts were associated with cognitive score. This was done by the linear mixed‐effect (LME) (Bates et al., 2014) model with cognitive function as outcome and age, sex, cell counts as fixed effects, and twin pairing as a random factor.

Before performing the statistical analyses, we first investigated the performance of three models, GCC (Murrell et al., 2016), LME, and kinship (Therneau, 2012) model by estimating their type I error rates using simulated random methylation data for one marker based on a standard normal distribution. Association of the simulated molecular data with cognitive function in the MADT cohort was assessed (*p* < 0.05 as significant) with type I error rates estimated as 0.052 for GCC, 0.054 for LME, and 0.055 for kinship model, upon 10,000 replicates. The models are generally unbiased, although the two linear models gave a slightly high type I error rate due to non‐optimal adjustment of twin correlation on cognition by the linear models.

### 2.1 Single CpG epigenome‐wide association studies

**FIGURE 1:**
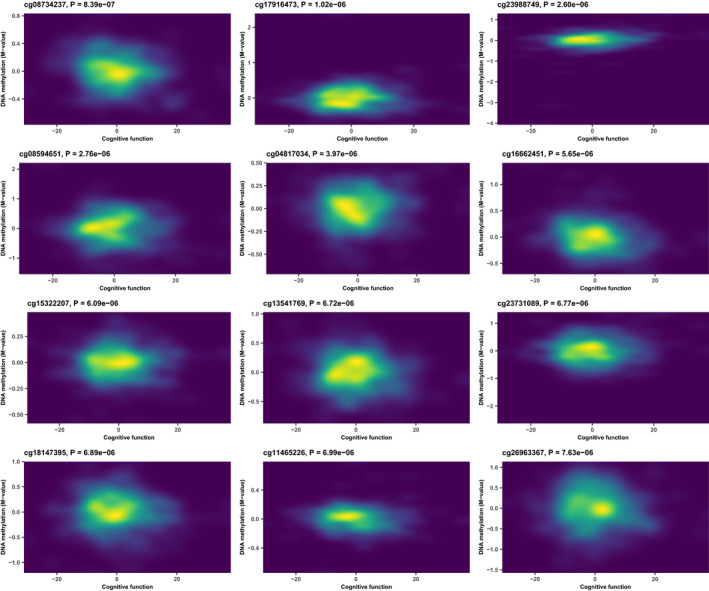
Density plot showing the relationship between DNA methylation M‐value and cognitive function for the top 12 significant CpGs in the final model

**FIGURE 2:**
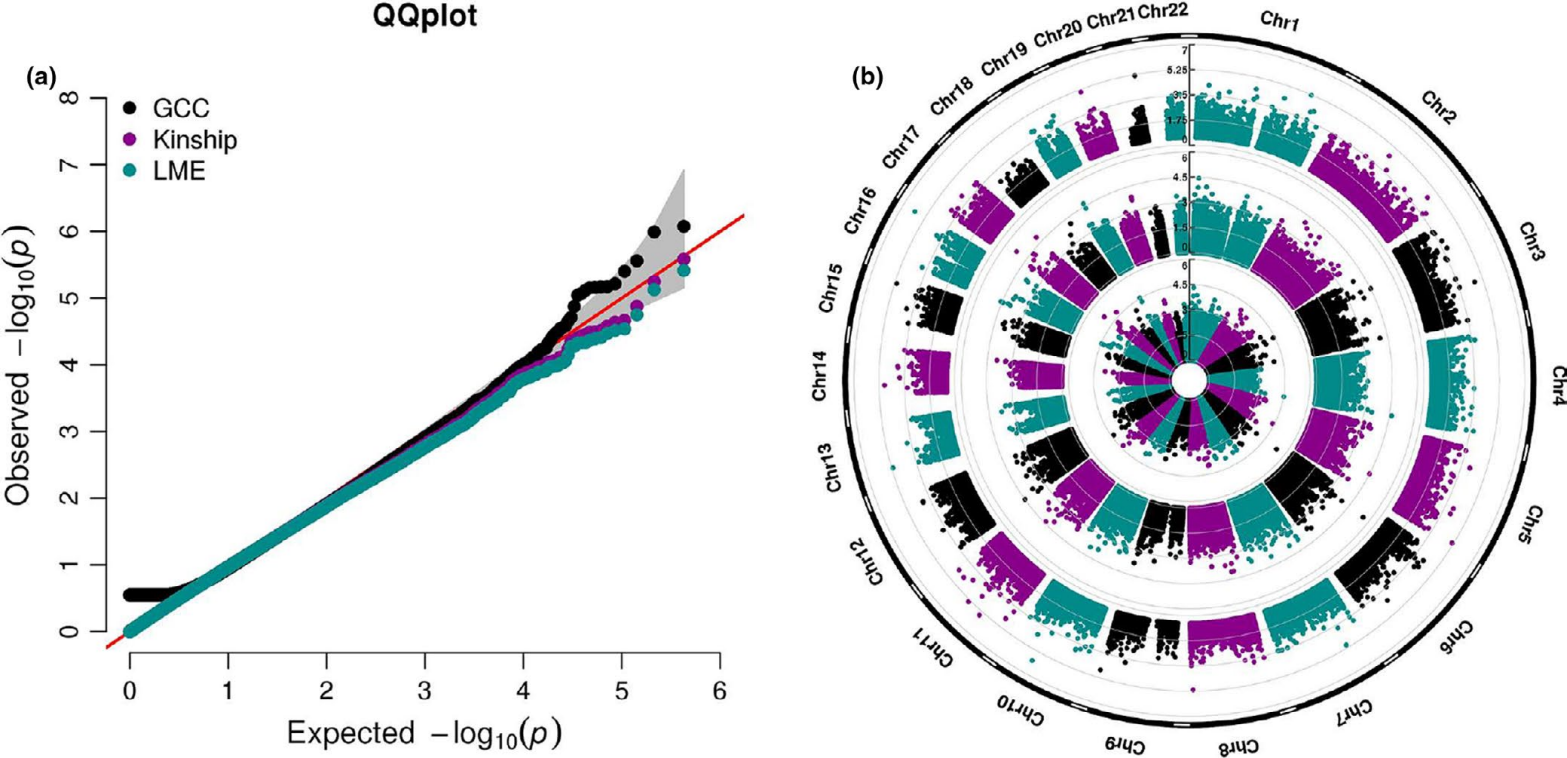
QQ plot and Manhattan plot of EWAS on cognitive function. (a) QQ plot comparing the performance of GCC, kinship, and LME models in EWAS data. (b) Circular Manhattan plot of GCC, Kinship, and LME based. The circular Manhattan plots from inner to outer are LME, Kinship, and GCC, respectively

**FIGURE 3:**
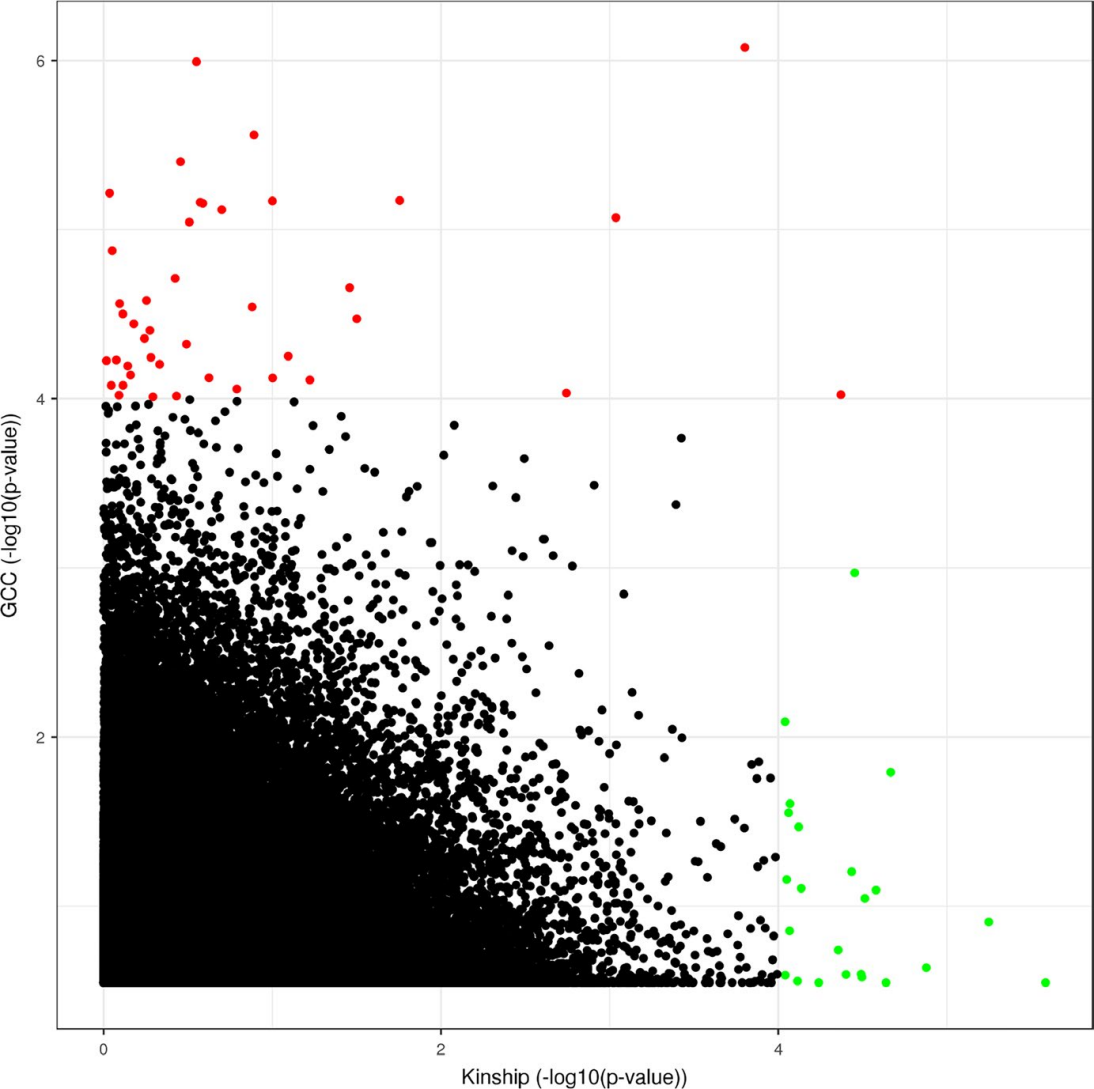
Scatter plot comparing the performance of CpGs in linear model to the GCC model. The x‐axis and y‐axis show −log10 (*p*‐value) from Kinship and GCC models, respectively

We applied the three models to examine the correlation between DNA methylation and cognitive function with methylation data in MADT cohort collected using the Infinium Human Methylation 450K BeadChip. After performing QC, 427,409 CpGs remained. The identified CpGs with *p* < 1e‐4 from GCC, Kinship, and LME were 42, 24, and 18. We chose Kinship results as a linear representative model (because of high consistency with LME) and GCC as a nonlinear method and combined the results by taking the minimum *p*‐values between them. After combining GCC results with linear model by taking the minimum *p*‐value between GCC and Kinship results, 65 CpGs were identified with *p* < 1e‐04, among them 14 CpGs with *p* < 1e‐05, 1 CpG with *p* < 1e‐06. No site reached genome‐wide significance defined as false discovery rate (FDR) < 0.05. Table 1 shows the top 30 CpGs captured by GCC (20 CpGs) and kinship model (10 CpGs). The top three CpGs are cg08734237 mapped to *KLHDC4* (*p* = 8.3871e‐07), cg17916473 mapped to *PAPSS2* (*p* = 1.0173e‐06), and cg23988749 mapped to *MRPS18B* (*p* = 2.5999e‐06). Table S2 shows the summary statistics results for the CpGs *p* < 0.05 from EWAS analysis. Figure 1 shows density plots between DNA methylation M‐value and cognitive function for the top 12 significant CpGs. Only two CpGs, cg23988749 and cg16662451, are captured by the linear model, while other 10 CpGs are significant for their nonlinear correlation with cognition. The QQ plot comparing the three models is illustrated in Figure 2a. We see that the upper tail CpGs from GCC deviate clearly from the diagonal line while many of those from the linear models were underestimated for their significance, reflecting poor model performances. The genomic inflation factors for GCC, Kinship, and LME were 0.99, 0.95, and 0.94, respectively. The circular Manhattan plot for the three models is displayed in Figure 2b. The top 30 CpGs captured by each of the models separately are shown in Tables S3–S5. GCC found more CpGs with low *p*‐value than the two linear models. Most of the CpGs in kinship and LME had the same order in the top list, but the kinship model reported lower *p*‐values. Figure 3 compares CpGs *p*‐values from the GCC EWAS with CpGs *p*‐values from the kinship model EWAS, with CpGs (*p* < 1e‐04) colored red if identified by GCC and green if identified by the kinship (linear) model. Similar comparisons of the GCC EWAS results with those of the LME model EWAS are shown in Figure S2. As the top CpGs from the linear models and GCC do not overlap, we ranked the CpGs by their lowest *p*‐values from either kinship or GCC to allow diverse patterns of correlation with cognitive function among top rank CpGs.

**TABLE 1:** Summary statistic of the top 30 CpGs from EWAS of cognitive function in final model from both GCC method and Kinship model

CpG	Ascore	*p*‐value GCC	Coef	*p*‐value Kinship	Gene	BP (hg19)	Genomic feature	*p*‐value	FDR
cg08734237	0.1616	8.3871e‐07	−0.0050	.0002	*KLHDC4*	16:87744948	Body, S_Shore	8.3871e‐07	0.2174
cg17916473	0.1605	1.0173e‐06	0.0017	.2816	*PAPSS2*	10:89419373	TSS200, Island	1.0173e‐06	0.2174
cg23988749	0	0.2832	0.0100	2.5999e‐06	*MRPS18B*	6:30585293	TSS200, Island	2.5999e‐06	0.2716
cg08594651	0.1542	2.7554e‐06	−0.0041	.1284	NA	11:47415397	NA, N_Shore	2.7554e‐06	0.2716
cg04817034	0.1519	3.9680e‐06	0.0010	.3502	*USP35*	11:77920577	Body, N_Shore	3.9680e‐06	0.2716
cg16662451	0.0328	0.1238	−0.0070	5.6459e‐06	*FBXW10*	17:18647507	5'UTR, NA	5.6459e‐06	0.2716
cg15322207	0.1491	6.0941e‐06	−0.00008	.9221	NA	1:211689087	NA, Island	6.0941e‐06	0.2716
cg13541769	0.1484	6.7241e‐06	−0.0038	.0176	*PRDM15*	21:43221684	Body, Island	6.7241e‐06	0.2716
cg23731089	0.1484	6.7682e‐06	0.0040	.0999	*EIF2C2*	8:141599208	Body, NA	6.7682e‐06	0.2716
cg18147395	0.1482	6.891e‐06	−0.0021	.2675	NA	13:30579100	NA, NA	6.8905e‐06	0.2716
cg11465226	0.1481	6.9891e‐06	−0.0009	.2582	*PRR7*	5:176882869	Body, Island	6.9891e‐06	0.2716
cg26963367	0.1476	7.6279e‐06	−0.0032	.1994	NA	15:89157841	NA, NA	7.6279e‐06	0.2717
cg04465201	0.1468	8.5029e‐06	0.0035	.0009	*EIF2S2*	20:32699025	Body, N_Shore	8.5029e‐06	0.2759
cg20497212	0.1464	9.0372e‐06	0.0012	.3105	*AOAH*	7:36672687	Body, NA	9.0372e‐06	0.2759
cg20482334	0.0127	0.2305	−0.0068	1.3245e‐05	*FASN*	17:80048531	Body, N_Shore	1.3245e‐05	0.2832
cg00744656	0.1438	1.3310e‐05	−0.0002	.8899	*FOXA1*	14:38063564	Body, N_Shore	1.3310e‐05	0.2832
cg01273125	0.1411	1.9438e‐05	−0.0021	.3767	*COBRA1*	9:140149675	TSS20, Island	1.9438e‐05	0.2832
cg20644253	0.0736	0.0162	0.0082	2.1548e‐05	*KIAA0892*	19:19431407	TSS15, Island	2.1548e‐05	0.2832
cg18191418	0.1402	2.2055e‐05	0.0043	.0348	NA	3:128336579	NA, Island	2.2055e‐05	0.2832
cg01182076	0.0345	0.2832	−0.0089	2.2968e‐05	*ODZ3*	4:183601697	Body, NA	2.2968e‐05	0.2832
cg16126079	0.1390	2.6257e‐05	−0.0007	.5574	*DNHD1*	11:6518322	TSS1500, NA	2.6257e‐05	0.2832
cg14100184	0.0433	0.0803	0.0095	2.6325e‐05	*GNG13*	16:851298	TSS1500, S_Shore	2.6325e‐05	0.2832
cg10238145	0.1387	2.7406e‐05	−0.0004	.8040	NA	2:114644207	NA, N_Shelf	2.7406e‐05	0.2832
cg07683636	0.1383	2.8700e‐05	−0.0018	.1317	*NHEJ1*	2:219940977	3'UTR, NA	2.8700e‐05	0.2832
cg00848394	0.0407	0.0898	0.0047	3.0661e‐05	*WDR51A*	3:52188768	TSS200, Island	3.0661e‐05	0.2832
cg07537095	0.1377	3.1549e‐05	0.0006	.7694	NA	11:116228957	NA, NA	3.1549e‐05	0.2832
cg14994060	0.0064	0.2627	−0.0045	3.1938e‐05	NA	5:134376489	NA, Island	3.1938e‐05	0.2832
cg04941278	0.0085	0.2523	−0.0085	3.2285e‐05	NA	12:3566096	NA,NA	3.2285e‐05	0.2832
cg09190408	0.1372	3.3643e‐05	−0.0031	.0316	*PPP1CA*	11:67170610	TSS1500,S_Shore	3.3643e‐05	0.2832
cg21112485	0.1079	0.0011	−0.0072	3.5209e‐05	*AGTRAP*	1:11808413	Body, NA	3.5209e‐05	0.2832

Abbreviations: Ascore, association score; FDR, false discovery rate.

To observe any difference in the genomic location of the CpGs with *p* < 0.05 with both positive (methylated) or negative (demethylated) correlation with cognitive function (as determined by the linear model estimates), we made a star plot in Figure S3a to show the distribution of methylated (green) and demethylated (blue) CpGs with cognitive function. Compared with the distribution of all CpGs in the 450k array (black), the demethylated CpGs are less distributed to the promoters. Figure S3b shows the frequency of CpGs with positive or negative correlations with cognition at each gene region. High proportions of demethylated CpGs are observed at gene body, 3′ UTR, and intergenic regions.

### 2.2 Biological pathway analysis

The total number of mapped genes (*p* < 0.05) was 27,413. These genes were used as input for over‐representation analysis of KEGG and REACTOME pathways as well as DisGeNET (human disease) implemented in *WebGestalt* (Liao et al., 2019). Table 2 shows the top 30 KEGG, REACTOME, and DisGeNET pathways with statistical significance (FDR < 5.88e‐04). Among them, there are cAMP, MAPK, Neurotrophin signaling pathways, Focal adhesion, Axon guidance, Ion homeostasis, Membrane Trafficking, and signal transduction from KEGG and REACTOME. Moreover, from DisGeNET diseases and phenotypes like Schizophrenia, Intellectual Disability, Mental Retardation, Low intelligence, Mental deficiency, Poor school performance, Dull intelligence, and Cognitive delay were overrepresented.

**TABLE 2:** The top 30 significant functional KEGG, REACTOME and DisGeNET pathways in WebGestalt

Gene set	Description	Size	Expect	Ratio	*p*‐value	FDR
R‐HSA‐5663202	Diseases of signal transduction	359	234.78	1.2267	2.5600e‐10	2.2946e‐7
R‐HSA‐5683057	MAPK family signaling cascades	275	179.85	1.2566	3.3039e‐10	2.2946e‐7
R‐HSA‐597592	Post‐translational protein modification	1268	829.26	1.1167	3.4078e‐10	2.2946e‐7
R‐HSA‐422475	Axon guidance	511	334.19	1.1850	7.6401e‐10	3.8583e‐7
hsa04024	cAMP signaling pathway	189	123.60	1.2945	1.8312e‐9	7.3981e‐7
R‐HSA‐5673001	RAF/MAP kinase cascade	235	153.69	1.2558	7.3300e‐9	2.4678e‐6
C0036341	Schizophrenia	944	623.27	1.1295	7.3798e‐10	2.5283e‐6
hsa04510	Focal adhesion	188	122.95	1.2769	1.9426e‐8	4.9050e‐6
hsa01521	EGFR tyrosine kinase inhibitor resistance	77	50.357	1.4099	3.5265e‐8	7.9150e‐6
R‐HSA‐9006934	Signaling by receptor tyrosine kinases	436	285.14	1.1714	1.5029e‐7	3.0358e‐5
C3714756	Intellectual disability	629	415.29	1.1390	1.2056e‐7	8.9901e‐5
C0025362	Mental retardation	545	359.83	1.1478	1.8369e‐7	8.9901e‐5
C0423903	Low intelligence	545	359.83	1.1478	1.8369e‐7	8.9901e‐5
C0917816	Mental deficiency	545	359.83	1.1478	1.8369e‐7	8.9901e‐5
C1843367	Poor school performance	545	359.83	1.1478	1.8369e‐7	8.9901e‐5
C4020876	Dull intelligence	545	359.83	1.1478	1.8369e‐7	8.9901e‐5
C0376634	Craniofacial abnormalities	136	89.793	1.2919	2.6809e‐7	9.5731e‐5
C0020534	Orbital separation excessive	219	144.59	1.2310	2.9492e‐7	9.5731e‐5
C1864897	Cognitive delay	541	357.19	1.1450	3.3871e‐7	9.5731e‐5
hsa01522	Endocrine resistance	93	60.821	1.3318	1.7661e‐6	2.4775e‐4
hsa04722	Neurotrophin signaling pathway	114	74.555	1.3011	1.8397e‐6	2.4775e‐4
hsa04015	Rap1 signaling pathway	201	131.45	1.2248	2.8739e‐6	3.6014e‐4
R‐HSA‐5578775	Ion homeostasis	54	35.315	1.4158	3.0309e‐6	3.6014e‐4
hsa05205	Proteoglycans in cancer	193	126.22	1.2280	3.2738e‐6	3.6613e‐4
R‐HSA‐2219528	PI3K/AKT signaling in cancer	95	62.129	1.3198	3.4438e‐6	3.6613e‐4
hsa05211	Renal cell carcinoma	62	40.547	1.3811	5.8214e‐6	5.8796e‐4

### 2.3 Analysis of differentially methylated regions

The EWAS final results from the combined CpGs of GCC and linear model were used for identification of DMRs, using *comb*‐*p* algorithm (Pedersen et al., 2012). In total, 15 DMRs with FDR < 0.05 were identified, which is shown in Table S6. The DMRs pattern has been depicted in Figure S4, which among them DMRs in Figure S4A–G, J are hyper‐methylated and DMRs in Figure S4H,O,I,K,L,N, M are hypo‐methylated with increasing cognitive function.

We used the 30 DMRs with *p* < 0.05 from *comb*‐*p* analysis as input in *GREAT* software (McLean et al., 2010) to identify their regulatory domain of the human genome (UCSC hg19). The Top functional clusters of biological process and molecular functions with (binomial *p* < 1e‐3) are shown in Table S7 and for a better visualization the directed acyclic graphs are shown in Figures S5 and S6. These biological processes include aggressive behavior, mating, female mating behavior, female receptivity, muscle contraction of the hindgut, hypermethylation of CpG island, as well as some metabolic processes such as alkene and ethylene. Additionally, top two identified molecular functions were neurohypophyseal hormone activity and oxytocin receptor binding.

### 2.4 Replication using Longitudinal Study of Aging Danish Twins data

As an effort for replication, we used the Longitudinal Study of Aging Danish Twins (LSADT) data to replicate the top 65 identified CpGs with *p* < 1e‐04 (Table S2). From LSADT, 9 CpGs were replicated which are shown in Table S8. Assuming the 65 discovered CpGs are random and type 1 error rate of 0.05, based on the binomial test the probability of having ≥9 replicates out of 65 is *p* = 0.00281. The replication result suggests that the identified 65 CpGs are extremely unlikely chance findings.

### 2.5 Further verification using gene expression data

Using gene expression data on the same MADT samples, we found 11 genes annotated to the 15 DMRs in Table S6 and investigated association of their expression levels with cognitive function using the combined GCC method and Kinship model. Among the 11 genes, 3 genes, *SMC1B*, *GABBR1*, and *HCG16* were confirmed associated with cognitive function with *p* < 0.05. In the gene expression data, two probes (*p* = 0.02, *p* = 0.27) were mapped to *GABBR1*, one probe (*p* = 0.02) to *SMC1B* and one probe (*p* = 0.04) to *HCG16*.

## 3 DISCUSSION

This study of DNA methylation data in twin samples indicates the strength of GCC in comparison with the traditional methods characterized by (1) ability to capture nonlinear correlation patterns missed by the linear models, (2) robustness in handling EWAS data on related samples such as twins, and (3) biologically meaningful annotations of identified markers.

Conventionally, linear relationship has been assumed in describing the association between a molecular marker and a phenotype of interest for simplicity in statistical modeling. In a genome‐wide association study (GWAS), this assumption translates to the additive genetic effects. The linear assumption suffers intrinsically from low efficiency in handling the biological complexity in the molecular regulation on phenotype expression, which cannot be simplified by just a linear model. This phenomenon has been clearly exemplified in a published microarray time course experiment analyzed using sophisticated parametric modeling (Murrell et al., 2016; Reshef et al., 2011). Instead of complex modeling, GCC provides an alternative assumption‐free approach inherently capable of capturing patterns of any non‐random relationship. Based on our findings, there were no top CpGs overlapping between GCC and linear models (Figure 3). In fact, linear models are defined and coded for linear relationships and GCC has to sacrifice power for some kinds of relationships to see other kinds. With this consideration, we suggest that GCC could with advantage be applied as a complement to the linear methods to ensure different patterns of correlation be captured with high efficiency. As shown in Table 1, the majority of the CpGs are detected by GCC presumably, because GCC is not limited to any predefined correlation pattern.

Although the single CpG EWAS did not find genome‐wide significant sites after correcting for multiple testing, the top rank CpGs were annotated to biologically meaningful genes and pathways.

In Table 1, *KLHDC4* mapped to the top significant CpG has been reported to be associated with Huntington Disease‐Like 2 (https://www.genecards.org/cgi‐bin/carddisp.pl?gene=KLHDC4), a disorder characterized partially by cognitive abnormalities. The second top significant *PAPSS2* gene is shown to be a novel longevity gene by expression profiling of genes within regions identified by a meta‐analysis GWAS of survival to age 90 (Yerges‐Armstrong et al., 2016). The third top significant *MRPS18B* gene is a mitochondrial gene. Mitochondrial are vital in providing energy for cells in the form of adenosine triphosphate (ATP) through oxidative phosphorylation (OXPHOS) (Lunnon et al., 2017). OXPHOS dysfunction can produce reactive oxygen species and oxidative stress leading to neuronal cell death in aging and in Alzheimer's disease (AD) brain (Devi et al., 2006).

Among the significant pathways in Table 2, it has been reported that the disturbance in signal transduction in brain cells causes the cognitive decline (Lo Vasco, 2018). Focal adhesion is involved in integrin adhesion, communication between the extracellular matrix and the actin cytoskeleton, and the regulation of many cell types. Loss of cell adhesion can lead to cell death and altered focal signaling has been linked to synaptic loss, which may cause AD (Caltagarone et al., 2007). There is evidence that axon guidance might play a role in some brain disorders such as Parkinson's and AD (Lesnick et al., 2007). MAPK signaling pathway regulates neuronal apoptosis, β‐ and γ‐secretase activity, and phosphorylation of *APP* and tau which has a role in the pathogenesis of AD (Kim & Choi, 2010). Neurotrophins, such as brain‐derived neurotrophic factor (BDNF), are essential regulators of neuronal survival and lower level of them are related to etiology of Alzheimer's and Huntington's diseases (Mitre et al., 2017). In the literature, there is also evidence of a direct or indirect link of Membrane trafficking, Ion homeostasis to cognitive function or other neurological disorders (Cuomo et al., 2015; Wang et al., 2013).

Analysis of DMRs based on the joint results of GCC and linear models had the power to detect 13 DMRs with FDR < 0.05 and two with 0.05 < FDR < 0.1. Among the genes harbored by these DMRs, three genes *SMC1B*, *GABBR1*, and *HCG16* were verified using gene expression analysis. The *GABBR1* gene is highly expressed in brain, and it is a main inhibitory in the central nervous. Dysfunctional GABA interneuron could represent a core pathophysiological mechanism underlying cognitive dysfunction in schizophrenia (Li et al., 2016; Xu & Wong, 2018). In the processes of learning and memory, changes in GABAergic function could be an important factor in both early and later stages of AD pathogenesis (Govindpani et al., 2017). The two other genes *SMC1B* and *HCG16* have not been reported yet to have a direct link to cognitive function. Most interestingly, functional annotation of the DMRs by *GREAT* reported highly relevant GO terms as shown in Table S7. Moreover, functional gene annotation analysis using *GREAT* from top identified DMRs revealed important biological and molecular functional clusters implicated in cognitive function. The link of neurohypophyseal hormone and oxytocin receptor binding with memory process and cognitive function has been already discussed (Fehm‐Wolfsdorf et al., 1984; Iovino et al., 2018; Strupp et al., 1983).

Oxytocin is produced in the hypothalamus and released into the circulation through the neurohypophyseal system (Ross & Young, 2009). It is shown that there is an impairing influence of memory by oxytocin (Fehm‐Wolfsdorf et al., 1984) and also it modulates social cognition and affiliative behavior in both sexes (Ross & Young, 2009). Oxytocin in brain also regulates anxiety‐behaviors, which has been shown to correlate the maternal aggressive behavior (Bosch et al., 2005).

In women, the rate of muscle contraction, especially the rate of velocity development (RVD), is predominantly associated with cognition, particularly in women with low muscle strength (Tian et al., 2019).

There is also evidence of the role of mating, female receptivity, hypermethylation of CpG island, and metabolic process in cognitive function (Haberman et al., 2012; Sharp et al., 2010; Smith et al., 2015).

In Table S8, the 9 replicated CpGs were annotated to genes including *EPHA8*, *PRDM15*, *PRR25*, *GPLD1*, *SLC1A3*, *DPYSL2*, and *NHEJ1*. Gu et al., (2005) reported that the *EphA8* receptor is capable of inducing a sustained increase in MAPK activity, thereby promoting neurite outgrowth in neuronal cells. *PRR25* is among the 171 age‐related differential expression genes (Lee & Lee, 2017). *GPLD1* gene has been reported to interact with Apolipoprotein A1 and *APOA4* (Deeg et al., 2001) and Lin et al., 2015 have shown that the low levels of *APOA1*, *APOC3*, and *APOA4* are associated with risk of AD. Importantly, the *GPLD1* is a candidate gene that modulates Aβ production (Seki et al., 2020). Wilmsdorff et al., (2013) reported that the increased *SLC1A3* expression indicates facilitated transport and may result in reduced glutamate neurotransmission. The gene *DPYSL2* is highly expressed in brain and associated with AD (https://www.genecards.org/cgi‐bin/carddisp.pl?gene=DPYSL2).

As an extra effort to characterize our identified CpGs, we tested the proportion of CpG‐SNPs in the top CpGs with *p *< 1e‐4 and compare it with the proportion of CpG‐SNPs from the whole 450k array using hypergeometric test. The proportion of CpG‐SNPs in our study is higher than the proportion of CpG‐SNPs in the 450k array with borderline significance (*p* = 0.05). This indicates that DNA methylation could be one of the ways that genetic variations influence cognitive aging as it is discussed in a literature (Shoemaker et al., 2010). Additionally, we found a significant overlap (*p* = 3.04e‐16) of top genes in our study with age‐related genes including *PAPSS2*, *MRPS18B*, *USP35*, *PRDM15*, *ODZ3*, *DNHD1*, *WDR51A*, *AGTRAP*, *OPCML*, *FLJ3281*, *CCDC102B*, *PRR16*, and *FGF12* in the Lothian Birth Cohorts (LBC) reported by Li et al., (2017). Also, a significant overlap (*p* = 4.54e‐12) of genes *KLHDC4*, *MRPS18B*, *USP35*, *EIF2C2*, *AOAH*, *COBRA1*, *OPCML*, and *SLC1A3* with age‐related genes in LSADT reported by Tan et al., (2016). The two genes *PRDM15* and *SLC1A3* are also among the list of genes in the replication analysis.

A limitation for the GCC method is that its association parameter has no direction compare to the linear regression models that report the coefficient regression with a direction of effect (+ or −). This is because the direction of effect cannot be determined in the case of nonlinear relationship. We propose that, for any significant CpG marker, its direction of effect be roughly determined by the sign of its coefficient from the linear model. This same idea has been implemented in R Bioconductor package *RTN* (https://www.bioconductor.org/packages/release/bioc/html/RTN.html) which uses a generalized correlation coefficient to estimate correlation between expression of a transcription factor gene and expression of a target gene, but uses the sign of Pearson's correlation (negative or positive) to determine the direction of correlation. Another possible limitation of this study is the use of DNA methylation for the whole blood rather than a brain tissue. However, studies have shown whether blood can be used as a proxy in methylation studies as the brain tissue is usually unavailable. They concluded that the methylation status of many CpGs in the blood mirror those in the brain (Aberg et al., 2013).

Dependent samples can lead to biased statistical assessment if not adjusted properly in association studies. In genome‐wide association analysis, the sub‐grouping of samples is responsible for inflated statistical significance which can be revealed by QQ plots. In this study, LME and kinship models were applied to account for the correlated structure in our twin pairs. As shown by the QQ plots in Figure 2a, the significance estimates by linear models were not inflated, suggesting that the random effects estimated in the linear models well captured the twin correlation. In practice, however, inflated significances are frequently observed due to model misspecification in parametric modeling. It is encouraging that the GCC as an assumption‐free method does not require any specific handling of the correlated twin samples in achieving unbiased assessments. This feature is especially valuable in association analysis of omics data for dealing with sample sub‐grouping due to population stratification or batch effect from non‐biological experimental variations.

## 4 CONCLUSION

Through applying both GCC and linear models to EWAS on cognitive function, we identified more and meaningful genes and pathways as well as DMRs that could implicate the cognitive performance and cognitive aging compared with restricting to the popular linear models. The assumption‐free GCC is also robust in dealing with correlated samples as in our twins. The intrinsic features of GCC enabled the identification of DNA methylation markers displaying diverse correlation patterns with cognitive function. Our results promote the use of GCC to complement the traditional linear models in EWAS studies for marker discovery and for biological interpretation.

## 5 MATERIALS AND METHODS

### 5.1 Population study

The study sample comprises 400 monozygotic (MZ) twins aged 56–80, including 220 male and 180 female pairs (Table S1) recruited by the Danish Twin Registry as a part of the middle‐aged Danish twin (MADT) study (https://pubmed.ncbi.nlm.nih.gov/31544734/). The whole blood samples were collected during 2008–11 in a follow‐up assessment. The mean and standard deviation of blood sampling age of individuals were 66.55 and 5.96, respectively. The general cognitive composite score comprised five cognitive tests, which included verbal fluency, attention, and working memory (digits forward and digits backward) and memory (immediate and delayed word recall). The cognitive test scores were standardized to mean 0 and standard deviation 1 and were summed to calculate the general cognitive composite scores. The cognitive function for each twin pair through plotting cognitive scores for twin 1 versus twin 2 is depicted in Figure S1. The details about sample collection have been described in detail elsewhere (McGue & Christensen, 2001).

Blood cell counts from blood leukocyte subtypes (monocytes, lymphocytes, basophils, neutrophils, eosinophils) were counted using a Coulter LH 750 Hematology Analyzer (Beckman Coulter).

The replication sample includes 206 twins comprised of 192 MZ twins and 14 dizygotic (DZ) twins (64 male and 142 female twins) (Table S1) recruited by the Danish Twin Registry as a part of the Longitudinal Study of Aging Danish Twins (LSADT) project. The mean and standard deviation of age at blood sampling were 79.30 and 4.01, respectively.

### 5.2 DNA methylation profiling

DNA methylation profiles in whole blood samples were analyzed using the Infinium HumanMethylation450 BeadChips (Illumina) containing 485,512 CpG sites across the human genome. The QC of the DNA methylation data was done based on two R packages: *MethylAid* (Van Iterson et al., 2014) and *minfi* (Aryee et al., 2014). Probes were removed from the analysis based on the following thresholds: zero signals, low bead count < 3 beads, *p*‐value detection > 0.01, cross‐reactive probes defined by Chen et al., 2013 and probes missing in > 5% samples, and sex‐chromosome and SNP probes were removed and the rest were imputed based on their median. This resulted in 427,409 CpGs left for analysis. Data normalization was done by functional normalization (Fortin et al., 2014). Before the statistical analysis, the methylation beta values were logit transformed to M values (log_2_ (β/(1−β)).

### 5.3 Statistical analysis

#### 5.3.1 Generalized correlation coefficient and linear regression models

We tested by fitting two linear regression models and GCC whether DNA methylation in whole blood is associated with cognitive function. In the linear models, both kinship model from *kinship2* R package (Therneau, 2012) and LME from *lme4* R package (Bates et al., 2014) were applied to find the association of DNA methylation levels with cognitive function. Before applying association testing, we adjusted for age, gender, and blood cell compositions. The kinship module calculates a kinship matrix and integrates it in the covariance matrix of the methylation data. The LME model corrects for correlation between twins in a pair by including twin pairing as a random effect in the model.

For GCC analysis, *Matie* R package was used (Murrell et al., 2016), which GCC was computed based on a ratio of maximum likelihoods for the marginal distribution and maximum weighted likelihoods for the joint distribution.

#### 5.3.2 Gene‐set enrichment analysis

The identified CpGs with *p* < 0.05 were annotated to genes and used as input for overrepresentation enrichment analysis (ORA) against the genes on the 450K array using the WEB‐based GEne SeT AnaLysis Toolkit (*WebGestalt*). Gene sets with FDR *q*‐value < 0.05 were reported as significant.

#### 5.3.3 Differentially methylated regions

In addition to single CpG based analysis, we also performed testing on multiple CpGs by extending our analysis to DMRs based on the results of single site testing. DMRs were detected using *comb*‐*p* tool developed by Pedersen et al. (2012). This method applies the Stouffer–Liptak approach to combine *p*‐values of the adjacent CpGs and reports significant regions after FDR adjustment. *Comb*‐*p* is a python library package (https://github.com/brentp/combined‐pvalues).

#### 5.3.4 Genomic regions enrichment analysis in GREAT

To analyze the functional significance of cis‐regulatory regions, we examined the regulatory domain of functional pathways from 30 DMRs *p* < 0.05 in the Genomic Regions Enrichment of Annotations Tool (*GREAT*).

#### 5.3.5 Replication analysis using LSADT data

For the detected top 65 significant methylation sites with *p* < 1e‐04, we performed a replication analysis using 450K DNA methylation data on an independent Danish twin cohort, the LSADT data. The association of DNA methylation with cognitive function was done on the GCC, Kinship, and LME models. And, the results from GCC and linear model were combined. Then, we used the binomial distribution to estimate the probability of having ≥*n* replicated CpGs out of 65 CpGs given the type one error rate of 0.05 and assuming the identified CpGs are by chance.

#### 5.3.6 Further verification by using gene expression data from MADT

Whole blood samples in gene expression data from MADT were collected, stabilized, and transported in PAXgene Blood RNA Tubes. Total RNA was extracted using the PAXgene Blood miRNA kit (Qiagen). The integrity and concentration of the isolated RNA were determined by the RNA 6000 Nano kit and a Bioanalyzer 2100 (Agilent Technologies). The raw intensity values were extracted from the scanned image files using Agilent Feature Extraction Software v. 10.7.3.1 (Agilent technologies).

Gene expression profiling was performed using the Agilent SurePrint G3 Human GE v2 8 × 60K Microarray. Sample labeling and array hybridization were carried out according to the “Two‐Color Microarray‐Based Gene Expression Analysis–Low Input Quick Amp Labeling” protocol (Agilent Technologies). Samples were labeled with Cy5, and the reference consisting of a pool of 16 samples was labeled with Cy3. Hybridization, washing, scanning, and quantification were performed according to the array manufacturer's recommendations.

The significant DMRs identified in *comb*‐*p* were mapped to genes, and these genes were verified in the aforementioned gene expression data from the same MADT cohort. The analysis was done by GCC, Kinship, and LME with adjustments on age, sex, and cell counts information similar to the methylation data. Then, the final assessment was done based on both GCC and Kinship models.

## CONFLICT OF INTEREST

The authors declare that they have no conflict of interest.

## AUTHOR CONTRIBUTIONS

AM, QT, and JH conceived the study. AM performed data analysis. AM and QT drafted the manuscript. All authors contributed in the manuscript, commented, and approved the submitted manuscript.

## ETHICS APPROVAL AND CONSENT TO PARTICIPATE

The MADT study was approved by the Regional Committees on Health Research Ethics for Southern Denmark (S‐VF‐19980072). Written informed consents were obtained from all participants in the Danish twin studies. Written informed consents were obtained from all LSADT participants and approved by the Regional Committees on Health Research Ethics for Southern Denmark (S‐VF‐20040241) and was conducted in accordance with the Helsinki II declaration.

## Supporting information

Supplementary Material

Table S2

Table S8

Table S9

Table S10

Table S11

Table S12

Table S13

## Data Availability

According to the Danish and EU legislations, transfer and sharing of individual‐level data require prior approval from the Danish Data Protection Agency and require that data sharing requests are dealt with on a case‐by‐case basis. However, we have provided information about sample in this study.
