# Supplementary material for: Novel DNA methylation marker discovery by assumption‐free genome‐wide association analysis of cognitive function in twins
Source: Aging Cell. 2021 Feb 2;20(2):e13293. doi: 10.1111/acel.13293 (PMC7884045; doi:10.1111/acel.13293)
Supplement: Supplementary file 1 — Supplementary Material [file ACEL-20-e13293-s001.docx]

**Table S1**. Descriptive statistics of the 400 monozygotic twins from MADT included in the discovery study and 206 twins (192 monozygotic twins and 14 dizygotic twins) from LSADT in the replication sample.

| **EWAS Variables** | **Male** | **Female** | **Total** |
| --- | --- | --- | --- |
| **MADT sample** | 220 | 180 | 400 |
| **Mean of age±**  **sd (min, max)** | 66.78±6.09(57.00,79.88) | 66.28±5.80(55.94,79.23) | 66.55±5.96(55.94,79.89) |
| **Mean of cognitive score±**  **sd (min, max)** | 44.04±9.81(11.68,84.93) | 47.67±8.98(24.98,75.39) | 45.67±9.61(11.68,84.93) |
| **LSADT sample** | 64 | 142 | 206 |
| **Mean of age±**  **sd (min, max)** | 79.21±3.94(74.33,87.57) | 79.34±4.05(73.43,90.67) | 79.30±4.01(73.43,90.67) |
| **Mean of cognitive score±**  **sd (min, max)** | 33.40±9.93(12.74,57.95) | 36.09±7.98(16.12,59.94) | 35.25±8.70(12.74,59.94) |


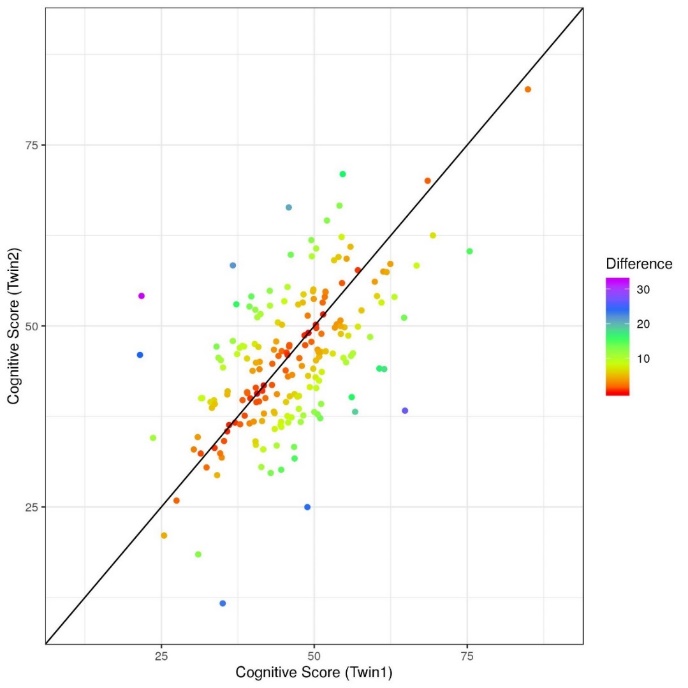


**Figure S1.** Scatter plot showing the correlation between twin pairs on their cognitive function (correlation coefficient = 0.6 and *p* < 2.2e-16). The X-axis shows cognitive score for twin 1 and Y-axis shows cognitive score for twin 2. The absolute value of the difference in cognitive function for twin 1 and twin2 is depicted in different colors.

**Table S3**. Summary statistic of the top 30 CpGs from EWAS of cognitive function in GCC model.

| **CpG** | **Ascore^a^** | **rawA^a^** | **P-value (unadjusted)** | **Gene** | **CHR** | **BP(hg19)** | **Genomic feature** | **FDR^b^** |
| --- | --- | --- | --- | --- | --- | --- | --- | --- |
| cg08734237 | 0.1616 | 0.0868 | 8.3871e-07 | *KLHDC4* | 16 | 87744948 | Body, S Shore | 0.2174 |
| cg17916473 | 0.1605 | 0.0857 | 1.0173e-06 | *PAPSS2* | 10 | 89419373 | TSS200, Island | 0.2174 |
| cg08594651 | 0.1542 | 0.0801 | 2.7554e-06 | *NA* | 11 | 47415397 | NA, N_Shore | 0.2832 |
| cg04817034 | 0.1519 | 0.078 | 3.9680e-06 | *USP35* | 11 | 77920577 | Body, N_Shore | 0.2832 |
| cg15322207 | 0.1491 | 0.0755 | 6.0941e-06 | *NA* | 1 | 211689087 | NA, Island | 0.2832 |
| cg13541769 | 0.1484 | 0.0749 | 6.7241e-06 | *PRDM15* | 21 | 43221684 | Body, Island | 0.2832 |
| cg23731089 | 0.1484 | 0.0749 | 6.7682e-06 | *EIF2C2* | 8 | 141599208 | Body, NA | 0.2832 |
| cg18147395 | 0.1482 | 0.0748 | 6.8905e-06 | *NA* | 13 | 30579100 | NA, NA | 0.2832 |
| cg11465226 | 0.1481 | 0.0747 | 6.9891e-06 | *PRR7* | 5 | 176882869 | Body, Island | 0.2832 |
| cg26963367 | 0.1476 | 0.0742 | 7.6279e-06 | *NA* | 15 | 89157841 | NA, NA | 0.2832 |
| cg04465201 | 0.1468 | 0.0736 | 8.5029e-06 | *EIF2S2* | 20 | 32699025 | Body, N_Shore | 0.2832 |
| cg20497212 | 0.1464 | 0.0732 | 9.0372e-06 | *AOAH* | 7 | 36672687 | Body, NA | 0.2832 |
| cg00744656 | 0.1438 | 0.0709 | 1.3310e-05 | *FOXA1* | 14 | 38063564 | Body, N_Shore | 0.2832 |
| cg01273125 | 0.1411 | 0.0686 | 1.9438e-05 | *COBRA1* | 9 | 140149675 | TSS200, Island | 0.2832 |
| cg18191418 | 0.1402 | 0.0679 | 2.2055e-05 | *NA* | 3 | 128336579 | NA, lsland | 0.2832 |
| cg16126079 | 0.139 | 0.0668 | 2.6257e-05 | *DNHD1* | 11 | 6518322 | TSS1500, NA | 0.2832 |
| cg10238145 | 0.1387 | 0.0666 | 2.7406e-05 | *NA* | 2 | 114644207 | NA, N_Shelf | 0.2832 |
| cg07683636 | 0.1383 | 0.0663 | 2.8700e-05 | *NHEJ1* | 2 | 219940977 | 3'UTR, NA | 0.2832 |
| cg07537095 | 0.1377 | 0.0657 | 3.1549e-05 | *NA* | 11 | 116228957 | NA, NA | 0.2832 |
| cg09190408 | 0.1372 | 0.0653 | 3.3643e-05 | *PPP1CA* | 11 | 67170610 | TSS1500, S_Shore | 0.2832 |
| cg24694691 | 0.1367 | 0.0649 | 3.6063e-05 | *LOC441897* | 1 | 111927153 | Body, NA | 0.2832 |
| cg13593391 | 0.136 | 0.0643 | 3.9469e-05 | *OPCML* | 11 | 132582488 | Body, NA | 0.2832 |
| cg23751922 | 0.1352 | 0.0637 | 4.4027e-05 | *CREG1* | 1 | 167523443 | TSS1500, S_Shore | 0.2832 |
| cg01300464 | 0.1346 | 0.0632 | 4.7538e-05 | *NA* | 19 | 56128874 | NA, S_Shore | 0.2832 |
| cg00972246 | 0.1334 | 0.0622 | 5.6051e-05 | *NA* | 1 | 228265972 | NA, N_Shelf | 0.2832 |
| cg18501202 | 0.1333 | 0.0621 | 5.7002e-05 | *NA* | 6 | 125855421 | NA, NA | 0.2832 |
| cg10869957 | 0.133 | 0.0619 | 5.9054e-05 | *NA* | 10 | 104605280 | NA, NA | 0.2832 |
| cg05941864 | 0.1329 | 0.0618 | 5.9634e-05 | *EPHA8* | 1 | 22893978 | Body, S_Shelf | 0.2832 |
| cg23562023 | 0.1326 | 0.0615 | 6.2552e-05 | *GPLD1* | 6 | 24429162 | 3'UTR,NA | 0.2832 |
| cg08783647 | 0.1324 | 0.0614 | 6.3957e-05 | *C6orf64* | 6 | 39081641 | Body, N_Shore | 0.2832 |

a Ascore: is the association score (including hyperbolic correction), rawA: is the association score before hyperbolic correction

from *matie* package in R.

b FDR: false discovery rate

**Table S4.** Summary statistic of the top 30 CpGs from EWAS of cognitive function in Kinship model.

| **CpG** | **Coef^a^** | **SE^a^** | **Z^a^** | **P-value**  **(unadjusted)** | **Gene** | **CHR** | | **BP(hg19)** | **Genomic feature** | **FDR^b^** |
| --- | --- | --- | --- | --- | --- | --- | --- | --- | --- | --- |
| cg23988749 | 0.01 | 0.0021 | 4.7001 | 2.5999e-06 | *MRPS18B* | | 6 | 30585293 | TSS200, Island | 0.9971 |
| cg16662451 | -0.007 | 0.0015 | -4.5392 | 5.6459e-06 | *FBXW10* | | 17 | 18647507 | 5'UTR,NA | 0.9971 |
| cg20482334 | -0.0068 | 0.0016 | -4.356 | 1.3245e-05 | *FASN* | | 17 | 80048531 | Body, N_Shore | 0.9971 |
| cg20644253 | 0.0082 | 0.0019 | 4.2482 | 2.1548e-05 | *KIAA0892* | | 19 | 19431407 | TSS1500,Island | 0.9971 |
| cg01182076 | -0.0089 | 0.0021 | -4.2339 | 2.2968e-05 | *ODZ3* | | 4 | 183601697 | Body, NA | 0.9971 |
| cg14100184 | 0.0095 | 0.0023 | 4.2031 | 2.6325e-05 | *GNG13* | | 16 | 851298 | TSS1500,S_Shore | 0.9971 |
| cg00848394 | 0.0047 | 0.0011 | 4.1685 | 3.0661e-05 | *WDR51A* | | 3 | 52188768 | TSS200, Island | 0.9971 |
| cg14994060 | -0.0045 | 0.0011 | -4.1592 | 3.1938e-05 | *NA* | | 5 | 134376489 | NA,Island | 0.9971 |
| cg04941278 | -0.0085 | 0.002 | -4.1567 | 3.2285e-05 | *NA* | | 12 | 3566096 | NA, NA | 0.9971 |
| cg21112485 | -0.0072 | 0.0017 | -4.1369 | 3.5209e-05 | *AGTRAP* | | 1 | 11808413 | Body, NA | 0.9971 |
| cg10299746 | 0.005 | 0.0012 | 4.1273 | 3.6706e-05 | *PRR25* | | 16 | 855300 | TSS200,N_shelf | 0.9971 |
| cg02266452 | 0.007 | 0.0017 | 4.1096 | 3.9634e-05 | *ANKRD42* | | 11 | 82905235 | TSS200, Island | 0.9971 |
| cg00491851 | 0.0091 | 0.0022 | 4.0933 | 4.2521e-05 | *FAM134C* | | 17 | 40760955 | Body, N_Shore | 0.9971 |
| cg15670475 | -0.0064 | 0.0016 | -4.0848 | 4.4107e-05 | *NA* | | 19 | 18905020 | NA, N_Shelf | 0.9971 |
| cg12398238 | 0.0062 | 0.0016 | 4.0227 | 5.7534e-05 | *TMUB2* | | 17 | 42264131 | TSS1500, Island | 0.9971 |
| cg06697694 | 0.0085 | 0.0022 | 3.966 | 7.3094e-05 | *MMP25* | | 16 | 3095980 | TSS1500, Island | 0.9971 |
| cg01863613 | -0.0066 | 0.0017 | -3.9577 | 7.5667e-05 | *CCDC55* | | 17 | 28443583 | TSS1500,N_Shoe | 0.9971 |
| cg19283806 | -0.0096 | 0.0024 | -3.954 | 7.6858e-05 | *CCDC102B* | | 18 | 66389420 | 5'UTR, NA | 0.9971 |
| cg11607927 | 0.0081 | 0.0021 | 3.9292 | 8.5223e-05 | *XPO1* | | 2 | 61765934 | TSS1500, Island | 0.9971 |
| cg12037509 | -0.0052 | 0.0013 | -3.928 | 8.5664e-05 | *DPYSL2* | | 8 | 26451955 | Body, NA | 0.9971 |
| cg13627598 | 0.0057 | 0.0015 | 3.9247 | 8.6843e-05 | *PCNXL3* | | 11 | 65387073 | Body, S_Shelf | 0.9971 |
| cg05796178 | 0.0047 | 0.0012 | 3.9184 | 8.9151e-05 | *LZTS1* | | 8 | 20110988 | Body, S_Shelf | 0.9971 |
| cg04364561 | -0.0048 | 0.0012 | -3.9137 | 9.0898e-05 | *FGF12* | | 3 | 192231892 | Body, N_Shore | 0.9971 |
| cg09361941 | 0.0042 | 0.0011 | 3.9131 | 9.1136e-05 | *C17orf68* | | 17 | 8128471 | 3'UTR, S_Shore | 0.9971 |
| cg10481417 | -0.0034 | 9e-04 | -3.8868 | 0.0001 | *CYP1A1* | | 15 | 75018528 | TSS1500, Island | 0.9971 |
| cg04227254 | -0.0059 | 0.0015 | -3.8814 | 0.0001 | *EHMT2* | | 6 | 31856329 | Body, N_Shore | 0.9971 |
| cg06332512 | -0.0066 | 0.0017 | -3.8755 | 0.0001 | *RAB40C* | | 16 | 670800 | Body, Island | 0.9971 |
| cg06078253 | -0.0069 | 0.0018 | -3.872 | 0.0001 | *CCDC132* | | 7 | 92860516 | TSS1500, NA | 0.9971 |
| cg04576042 | -0.0128 | 0.0033 | -3.8687 | 0.0001 | *ZMYM1* | | 1 | 35545182 | 5'UTR, Island | 0.9971 |
| cg02836479 | 0.0043 | 0.0011 | 3.8677 | 0.0001 | *UQCRC2* | | 16 | 21964489 | TSS200, NA | 0.9971 |

a coef: coefficient from the association test, SE: standard error, Z: z statistic

b FDR: false discovery rate

**Table S5.** Summary statistic of the top 30 CpGs from EWAS of cognitive function in LME model.

| **CpG** | **Coef^a^** | **SE^a^** | **t-value** | **P-value (unadjusted)** | **Gene** | **CHR** | **BP(hg19)** | **Genomic feature** | **FDR^b^** |
| --- | --- | --- | --- | --- | --- | --- | --- | --- | --- |
| cg23988749 | 0.01 | 0.0021 | 4.7001 | 3.8558e-06 | *MRPS18B* | 6 | 30585293 | TSS200,Island | 0.9971 |
| cg16662451 | -0.007 | 0.0015 | -4.5394 | 7.4743e-06 | *FBXW10* | 17 | 18647507 | 5'UTR,NA | 0.9971 |
| cg20482334 | -0.0068 | 0.0016 | -4.356 | 1.7809e-05 | *FASN* | 17 | 80048531 | Body,N_Shore | 0.9971 |
| cg20644253 | 0.0082 | 0.0019 | 4.2441 | 2.8968e-05 | *KIAA0892* | 19 | 19431407 | TSS1500,Island | 0.9971 |
| cg01182076 | -0.0089 | 0.0021 | -4.2314 | 3.0572e-05 | *ODZ3* | 4 | 183601697 | Body,NA | 0.9971 |
| cg14100184 | 0.0095 | 0.0023 | 4.2031 | 3.3791e-05 | *GNG13* | 16 | 851298 | TSS1500,S_Shore | 0.9971 |
| cg00848394 | 0.0047 | 0.0011 | 4.1685 | 3.9271e-05 | *WDR51A* | 3 | 52188768 | TSS200,Island | 0.9971 |
| cg14994060 | -0.0045 | 0.0011 | -4.163 | 4.0712e-05 | *NA* | 5 | 134376489 | NA,Island | 0.9971 |
| cg04941278 | -0.0085 | 0.002 | -4.1526 | 4.2443e-05 | *NA* | 12 | 3566096 | NA,NA | 0.9971 |
| cg10299746 | 0.005 | 0.0012 | 4.1273 | 4.6758e-05 | *PRR25* | 16 | 855300 | TSS200,N_Shelf | 0.9971 |
| cg21112485 | -0.0072 | 0.0017 | -4.1287 | 4.6844e-05 | *AGTRAP* | 1 | 11808413 | Body,NA | 0.9971 |
| cg02266452 | 0.007 | 0.0017 | 4.1159 | 4.6871e-05 | *ANKRD42* | 11 | 82905235 | TSS200,Island | 0.9971 |
| cg00491851 | 0.0091 | 0.0022 | 4.0933 | 5.2969e-05 | *FAM134C* | 17 | 40760955 | Body,N_Shore | 0.9971 |
| cg15670475 | -0.0064 | 0.0016 | -4.0848 | 5.3300e-05 | *NA* | 19 | 18905020 | NA,S_Shelf | 0.9971 |
| cg12398238 | 0.0062 | 0.0016 | 4.0227 | 7.1169e-05 | *TMUB2* | 17 | 42264131 | TSS1500,Island | 0.9971 |
| cg06697694 | 0.0085 | 0.0022 | 3.966 | 8.7885e-05 | *MMP25* | 16 | 3095980 | TSS1500,Island | 0.9971 |
| cg01863613 | -0.0066 | 0.0017 | -3.9577 | 9.3241e-05 | *CCDC55* | 17 | 28443583 | TSS1500,N_Shore | 0.9971 |
| cg19283806 | -0.0096 | 0.0024 | -3.954 | 9.4698e-05 | *CCDC102B* | 18 | 66389420 | 5'UTR,NA | 0.9971 |
| cg11607927 | 0.0081 | 0.0021 | 3.9292 | 0.0001 | *XPO1* | 2 | 61765934 | TSS1500,Island | 0.9971 |
| cg12037509 | -0.0052 | 0.0013 | -3.928 | 0.0001 | *DPYSL2* | 8 | 26451955 | Body,NA | 0.9971 |
| cg13627598 | 0.0057 | 0.0015 | 3.9247 | 0.0001 | *PCNXL3* | 11 | 65387073 | Body,S_Shelf | 0.9971 |
| cg04364561 | -0.0048 | 0.0012 | -3.9136 | 0.0001 | *FGF12* | 3 | 192231892 | Body,N_Shore | 0.9971 |
| cg05796178 | 0.0047 | 0.0012 | 3.9238 | 0.0001 | *LZTS1* | 8 | 20110988 | Body,S_Shelf | 0.9971 |
| cg09361941 | 0.0042 | 0.0011 | 3.9131 | 0.0001 | *C17orf68* | 17 | 8128471 | 3'UTR,S_Shore | 0.9971 |
| cg10481417 | -0.0034 | 9e-04 | -3.8868 | 0.0001 | *CYP1A1* | 15 | 75018528 | TSS1500,Island | 0.9971 |
| cg04227254 | -0.0059 | 0.0015 | -3.8814 | 0.0001 | *EHMT2* | 6 | 31856329 | Body,N_Shore | 0.9971 |
| cg06078253 | -0.0069 | 0.0018 | -3.872 | 0.0001 | *CCDC132* | 7 | 92860516 | TSS1500,NA | 0.9971 |
| cg04576042 | -0.0128 | 0.0033 | -3.8689 | 0.0001 | *ZMYM1* | 1 | 35545182 | 5'UTR,Island | 0.9971 |
| cg06332512 | -0.0066 | 0.0017 | -3.8755 | 0.0001 | *RAB40C* | 16 | 670800 | Body,Island | 0.9971 |
| cg02836479 | 0.0043 | 0.0011 | 3.8679 | 0.0001 | *UQCRC2* | 16 | 21964489 | TSS200,NA | 0.9971 |

^a^ coef: coefficient from the association test, SE: standard error, t-value: t statistic

^b^ FDR: false discovery rate

**Figure S2**. Scatter plot comparing the performance of CpGs in linear model to the GCC model. The x-axis and y-axis show -log10(p-value) from LME and GCC models respectively.

**Figure S3.** Star plot showing **(a)** proportion of methylated (green) and demethylated (blue) CpGs in association with cognitive function over the gene regions and **(b)** region-specific proportion of methylated and demethylated CpGs with cognitive function by gene regions.

**Table S6**. Annotation to the significant DMRs (FDR < 0.1) from comb-p.

| **Chrom** | **Start** | **End** | **#Probes** | **Region-Stouffer-Liptak-p-value** | **FDR^a^** | **Genes** |
| --- | --- | --- | --- | --- | --- | --- |
| chr7 | 56160687 | 56160894 | 6 | 1.409e-07 | 0.0003 | *PHKG1* |
| chr15 | 49462287 | 49462515 | 5 | 2.457e-07 | 0.0005 | *GALK2* |
| chr6 | 30881560 | 30881894 | 18 | 3.801e-07 | 0.0005 | *GTF2H4* |
| chr13 | 47472200 | 47472361 | 6 | 1.998e-07 | 0.0005 |  |
| chr22 | 45809244 | 45809403 | 5 | 1.084e-06 | 0.0029 | *SMC1B* |
| chr6 | 29595491 | 29595662 | 4 | 2.126e-06 | 0.0053 | *GABBR1* |
| chr8 | 141599141 | 141599209 | 3 | 1.616e-06 | 0.0101 | *AGO2* |
| chr21 | 43221559 | 43221685 | 2 | 3.053e-06 | 0.0103 | *PRDM15* |
| chr17 | 28443555 | 28443641 | 4 | 2.392e-06 | 0.0118 |  |
| chr12 | 51566756 | 51566915 | 6 | 5.97e-06 | 0.0159 | *TFCP2* |
| chr6 | 48036584 | 48036677 | 7 | 3.665e-06 | 0.0167 | *PTCHD4* |
| chr20 | 3052115 | 3052275 | 7 | 6.807e-06 | 0.0180 |  |
| chr11 | 13983784 | 13983894 | 6 | 1.041e-05 | 0.0396 |  |
| chr2 | 240181892 | 240181949 | 3 | 6.358e-06 | 0.0466 | *HDAC4* |
| chr6 | 28956259 | 28956427 | 10 | 2.926e-05 | 0.0718 | *HCG16* |

^a^ FDR: false discovery rate

**Figure S4.** The patterns of top 15 differential methylation regions (DMRs).

**Table S7.** Top functional clusters biological process and molecular functions identified by GREAT.

| **GO Type** | **Term Name** | **Binom Raw P-Value** | **Binom FDR Q-Val** | **Binom Fold Enrichment** | **Binom Observed Region Hits** |
| --- | --- | --- | --- | --- | --- |
| MF^a^ | neurohypophyseal hormone activity | 6.65e-7 | 2.81e-3 | 1703.899 | 2 |
| MF | oxytocin receptor binding | 3.86e-4 | 8.13e-1 | 2593.211 | 1 |
| BP^b^ | maternal aggressive behavior | 3.72e-5 | 4.89e-1 | 227.228 | 2 |
| BP | mating | 2.41e-4 | 1.00 | 24.954 | 3 |
| BP | positive regulation of hindgut contraction | 3.86e-4 | 1.00 | 2593.211 | 1 |
| BP | hyperosmotic salinity response | 4.06e-4 | 1.00 | 68.361 | 2 |
| BP | ethylene metabolic process | 4.49e-4 | 1.00 | 2228.465 | 1 |
| BP | dibenzo-p-dioxin catabolic process | 4.49e-4 | 1.00 | 2228.465 | 1 |
| BP | regulation of female receptivity | 5.92e-4 | 1.00 | 56.523 | 2 |
| BP | female mating behavior | 6.01e-4 | 9.88e-1 | 56.115 | 2 |
| BP | hypermethylation of CpG island | 7.85e-4 | 1.00 | 1272.814 | 1 |
| BP | cellular alkene metabolic process | 9.66e-4 | 1.00 | 1035.224 | 1 |
| BP | aggressive behavior | 9.78e-4 | 1.00 | 43.845 | 2 |

^a^ MF: Molecular Function

^b^ BP: Biological pathway

**Figure S5.** A directed acyclic graph of GO biological process for top 30 DMRs with *p* < 0.05.

**Figure S6.** A directed acyclic graph of GO Molecular function from top 30 DMRs with *p* < 0.05.
